# Association of fish and meat consumption with non-alcoholic fatty liver disease: Guangzhou Biobank Cohort Study

**DOI:** 10.1186/s12889-023-17398-6

**Published:** 2023-12-06

**Authors:** Rui Zhen Wang, Wei Sen Zhang, Chao Qiang Jiang, Feng Zhu, Ya Li Jin, Lin Xu

**Affiliations:** 1 School of Public Health, Sun Yat-Sen University, 74 Zhongshan 2nd Road, Guangzhou, 510080 China; 2 Guangzhou Twelfth People’s Hospital, No.1 Tianqiang Road, Guangzhou, 510620 China; 3 School of Public Health, the University of Hong Kong, Hong Kong, China; 4 Institute of Applied Health Research, University of Birmingham, Birmingham, UK

**Keywords:** Non-alcoholic fatty liver disease, Fish consumption, Meat consumption, Cross-sectional study

## Abstract

**Background:**

Non-alcoholic fatty liver disease (NAFLD) is the most common chronic liver disease. Unhealthy dietary habit is one of major risk factors of NAFLD. However, the associations between specific types of fish and meat consumption and NAFLD remain inconclusive. We explored the associations of fish and meat consumption with NAFLD risk in middle-aged and older Chinese.

**Methods:**

We collected information on 1,862 participants aged 50 years or older from Guangzhou Biobank Cohort Study in 2009 to 2010. Fish and meat consumption was assessed using a validated food-frequency questionnaire. NAFLD was diagnosed by ultrasound. Multivariable logistic regression was used to examine the associations of fish and meat consumption with the presence of NAFLD.

**Results:**

The average age was 61.0 (standard deviation = 6.5) years for the participants, 50.2% were women, and 37.2% were diagnosed with NAFLD. After adjusting for age, sex, education, family income, occupation, smoking status, drinking status, physical activity and several metabolic traits, compared with 0 serving/week (one serving = 50 g), fatty fish consumption of ≥ 3 servings/week showed higher odds of NAFLD (odds ratio (OR) and 95% confidence interval (CI): 1.64 (1.12, 2.39)). The highest (≥ 11 servings/week of red meat and poultry; ≥ 3 servings/week of processed meat) versus the lowest (0–3 servings/week of red meat and poultry; 0 serving/week of processed meat) consumption of all other types of meats, including red meat, poultry and processed meat, showed no association with NAFLD (1.17 (0.75, 1.81), 1.02 (0.42, 2.50) and 0.85 (0.50, 1.45), respectively). Aquatic and sea food, and red meat had negative indirect effects on NAFLD via systolic blood pressure and/or high-density lipoprotein cholesterol. Processed meat had positive indirect effects on NAFLD via body mass index, waist circumference, fasting plasma glucose and triglycerides.

**Conclusion:**

High consumption of fatty fish was associated with higher NAFLD risk. Our results, if causal, provide evidence that limiting consumption of fatty fish can be considered as part of NAFLD lifestyle prevention and treatment.

**Supplementary Information:**

The online version contains supplementary material available at 10.1186/s12889-023-17398-6.

## Background

Non-alcoholic fatty liver disease (NAFLD) is the most common chronic liver disease worldwide, with a prevalence of approximately 25% [1]. NAFLD is defined as the accumulation of hepatic fat more than 5% and not caused by excessive alcohol consumption, use of hepatotoxic medications or other established liver diseases [2, 3]. NAFLD has been associated with increased risk of diabetes, cardiovascular disease and hepatocellular carcinoma [4–6]. However, there is a lack of effective treatment of NAFLD nowadays [7].

Unhealthy diet is a major modifiable factor of NAFLD. Different dietary composition may have different effects on NAFLD [8–11]. Fish contains various essential nutrients, such as polyunsaturated fatty acids, protein, selenium and vitamin D [12]. And of fishes, fatty fish contains more omega-3 polyunsaturated fatty acids (n-3 PUFAs), which may be beneficial to liver fat accumulation, despite the effects of n-3 PUFAs supplementation on liver histology features of NAFLD were not conclusive [13, 14]. Another important source of energy and essential nutrients, such as protein, iron, zinc, selenium and B vitamins, is meat consumption [15]. Meat consumption per capita has been increasing worldwide in the past decades [16]. In 2015, Chinese adults consumed meat at an average level of 94.0 g/day, which was much higher than that recommended by the Chinese Dietary Guidelines (75.0 g/day) (2016) [17]. As meat is also a major source of cholesterol, saturated fatty acids, as well as other potential harmful chemicals (i.e., heme iron and heterocyclic aromatic amines) [16], high meat consumption has also been associated with higher oxidative stress [18], insulin resistance (IR) [19] and type 2 diabetes [15]. These diseases have similar pathogenesis with NAFLD [20, 21], and therefore similar concerns regarding meat consumption may also apply to NAFLD.

Moreover, previous results on the association of fish and meat consumption with NAFLD have been inconsistent in terms of direction and magnitude of the associations [22–26]. Data on specific types of fish and meat consumption with NAFLD are largely scarce [22–26]. Therefore, we explored the associations of specific types of fish and meat consumption with NAFLD in middle-aged and older adults in southern China, taking advantage of thorough data collected in a well-established on-going cohort, the Guangzhou Biobank Cohort Study (GBCS).

## Methods

### Study design and population

This is a cross-sectional study using data collected during the first follow-up examination of the GBCS from 2009 to 2010. The Guangzhou Medical Ethics Committee of the Chinese Medical Association approved the study. All participants provided written informed consent before participation. Details of the GBCS have been reported previously [27, 28].

Of 30,430 participants recruited at baseline in 2003–2008, due to budget constraints, we focused on a subset who came back for the first follow-up examination from March 16, 2009, to September 15, 2010. From this subset, we implemented following specific criteria to select 2,008 participants for the NAFLD examination, including the availability during the examination period, willingness to undergo NAFLD examination, and complete data from initial enrollment and baseline measurements. Of the 2,008 participants, we excluded 143 participants with missing information on variables of interest and three participants who were heavy alcohol users, yielding 1,862 participants being included in the data analysis. Heavy alcohol use was defined as consuming ethanol more than 210 gram per week in men or 140 gram per week in women in the past twelve months. Details in assessment of alcohol use in the GBCS have been reported previously [29, 30].

### Outcome assessment

The study outcome was the presence of NAFLD. NAFLD was assessed by abdominal ultrasonography (Philips, iU22) and diagnosed according to the established criteria from the Chinese Society of Hepatology in 2010 [31]. Compared to liver histology, ultrasound demonstrates an 85% sensitivity and 94% specificity in detecting moderate to severe fatty liver conditions [32, 33]. The ultrasound examination was conducted in the Guangzhou Twelfth People’s Hospital. Ultrasonographic examination was performed by an experienced radiologist using a real-time scanner equipped with a convex-array probe, who was blinded to other information of the participants. Results were double-checked by another senior radiologist to ensure an unbiased evaluation.

### Exposure assessment

Fish and meat consumption was measured using a validated food-frequency questionnaire (FFQ) by trained nurses in a face-to-face review [27, 34]. For each meat item, average frequency of consumption over the last seven days and amount of consumption each time were assessed, with portion sizes explained using a catalogue of pictures of individual food portions. The following response options for frequencies were provided to participants: never, not over the past week, 1–2 times/week, 3–4 times/week, 5–6 times/week, once/day, twice/day and 3 + times/day. Then the average amount each time consumed was asked by providing the following options: less than one portion, one portion, 2–3 portions and 4 portions or more, with one portion equaling to 50 g. Each type of fish and meat consumption was calculated by multiplying frequency by type-specific amount using the median value of each response option, and expressed as servings per week, with one serving equaling 50 g. Fatty fish, defined as n-3 fatty acid (FA) (eicosapentaenoic acid (EPA) and docosahexaenoic acid (DHA)) values ≥ 0.5 g/100 g tissue [35], consisted of mackerel, finless eel, snakehead, sea eel, salmon and sardine. Other fishes included grass carp, bullhead, bass and sequoia fish, and aquatic and sea food consisted of shrimp, crab, scallops and mussel. Red meat was composed of pork, beef and mutton. Poultry included roast duck, steamed chicken, plain chicken and stewed pigeon. Processed meat included cured meat and salted fish.

### Potential confounders and mediators

Potential confounders considered included the following variables: age (years), sex (women and men), education (primary or below, middle school, and college or above), family income (< 10,000, 10,000–29,999, 30,000–49,999, ≥ 50,000 CNY/year, and don’t know), occupation (manual, non-manual, and other), alcohol drinking (never and ever), smoking status (never, former, and current) and physical activity assessed by International Physical Activity Questionnaire (low, moderate, and active) [36]. All participants had assessments for anthropometrics (height, weight, waist circumference and blood pressure) and biomarkers (fasting plasma glucose (FPG), total cholesterol (TC), triglycerides (TG), high-density lipoprotein cholesterol (HDL-C) and low-density lipoprotein cholesterol (LDL-C) by trained nurses. Body mass index (BMI) was calculated by weight in kilograms divided by height squared in meters. These anthropometrics and biomarkers were considered as potential mediators.

### Statistical analysis

Continuous variables were expressed as mean (standard deviation, SD) or median (interquartile range, IQR), and categorical variables were presented as frequencies (percentages). For categorial variables such as sex, education, family income, occupation, drinking status, smoking status, physical activity, fish and meat consumption, we used chi-square test to determine the differences across NAFLD groups. Continuous variables (i.e., age, BMI, waist circumference, systolic blood pressure (SBP), diastolic blood pressure (DBP), FPG, TG, TC, HDL-C and LDL-C) were analyzed using analysis of variance (ANOVA). Similarly, we used chi-square and ANOVA tests to compare characteristics across different groups of fish and meat consumption. Additionally, for specific cases where these tests were not applicable, we utilized the Fisher’s exact probability test. Multivariable logistic regression was used to assess the associations of fish and meat consumption with NAFLD with adjustment for potential confounders, yielding odds ratios (ORs) and 95% confidence intervals (CIs). We also performed a mediation analysis using bootstrapping method to test whether there were indirect effects of fish and meat consumption on NAFLD via metabolic traits (i.e., BMI, waist circumference, FPG, SBP, DBP, TG, TC, HDL-C and LDL-C). For these traits which were not significant mediating factors, they were further adjusted in the multivariable logistic regression models. Stata version 16.0 was used for data analysis. All *P* values were 2-sided, and *P* < 0.05 was considered statistically significant.

## Results

### Description of study population

Of 1,862 participants, 50.16% were women. The mean (standard deviation) age was 60.90 (6.49) years and the prevalence of NAFLD was 37.22%. Table 1 shows that men and smokers were more likely to have NAFLD (all *P* < 0.001). Participants with NAFLD had higher BMI, waist circumference, SBP, DBP, FPG and TG, but lower HDL-C (all *P* < 0.001) (Table 1).

**Table 1:** Characteristics of study sample by NAFLD on 1,862 participants of the Guangzhou Biobank Cohort Study

	Total (n = 1,862)	NAFLD		*P*
		Without (n = 1,169)	With (n = 693)	
Age (years), mean (SD)	60.90 (6.49)	60.77 (6.50)	61.12 (6.46)	0.26
Sex, n (%)				< 0.001
Women	934 (50.16)	633 (54.15)	301 (43.43)	
Men	928 (49.84)	536 (45.85)	392 (56.57)	
Education, n (%)				0.35
Primary or below	664 (35.66)	424 (36.27)	240 (34.63)	
Middle school	979 (52.58)	617 (52.78)	362 (52.24)	
College or above	219 (11.76)	128 (10.95)	91 (13.13)	
Family income (CNY/year), n (%)				0.05
< 10,000	23 (1.24)	12 (1.03)	11 (1.59)	
10,000–29,999	209 (11.22)	148 (12.66)	61 (8.80)	
30,000–49,999	526 (28.25)	339 (29.00)	187 (26.98)	
≥ 50,000	238 (12.78)	144 (12.32)	94 (13.56)	
Don’t know	866 (46.51)	526 (45.00)	340 (49.06)	
Occupation, n (%)				0.58
Manual	1,025 (55.05)	653 (55.86)	372 (53.68)	
Non-manual	481 (25.83)	293 (25.06)	188 (27.13)	
Other	356 (19.12)	223 (19.08)	133 (19.19)	
Drinking status, n (%)				0.51
Never	511 (27.44)	327 (27.97)	184 (26.55)	
Ever	1,351 (72.56)	842 (72.03)	509 (73.45)	
Smoking status, n (%)				< 0.001
Never	1,289 (69.23)	843 (72.11)	446 (64.36)	
Former	258 (13.86)	135 (11.55)	123 (17.75)	
Current	315 (16.92)	191 (16.34)	124 (17.89)	
Physical activity, n (%)				0.11
Low	13 (0.70)	6 (0.51)	7 (1.01)	
Moderate	479 (25.73)	286 (24.47)	193 (27.85)	
Active	1,370 (73.58)	877 (75.02)	493 (71.14)	
BMI (kg/m^2^), mean (SD)	23.85 (3.46)	22.52 (2.76)	26.09 (3.36)	< 0.001
Waist circumference (cm), mean (SD)	83.36 (9.34)	79.57 (7.62)	89.75 (8.44)	< 0.001
SBP (mmHg), mean (SD)	130.58 (19.63)	127.61 (19.26)	135.59 (19.25)	< 0.001
DBP (mmHg), mean (SD)	73.40 (10.26)	71.57 (9.73)	76.51 (10.40)	< 0.001
FPG (mmol/L), median (IQR)	5.09 (4.72, 5.57)	4.97 (4.67, 5.40)	5.31 (4.88, 5.92)	< 0.001
TC (mmol/L), mean (SD)	5.67 (1.13)	5.69 (1.10)	5.64 (1.18)	0.36
TG (mmol/L), median (IQR)	1.45 (1.04, 2.11)	1.26 (0.94, 1.80)	1.85 (1.30, 2.73)	< 0.001
HDL-C (mmol/L), mean (SD)	1.31 (0.30)	1.37 (0.31)	1.22 (0.25)	< 0.001
LDL-C (mmol/L), mean (SD)	3.38 (0.90)	3.37 (0.88)	3.39 (0.93)	0.60

NAFLD, non-alcoholic fatty liver disease; CNY, Chinese Yuan (US$1 = 7 CNY); SD, standard deviation; IQR, interquartile range; BMI, body mass index; SBP, systolic blood pressure; DBP, diastolic blood pressure; FPG, fasting plasma glucose; TC, total cholesterol; TG, triglycerides; HDL-C, high-density lipoprotein cholesterol; LDL-C, low-density lipoprotein cholesterol

In addition, sample characteristics by different types of fish and meat consumption were shown in the Supplementary Tables 1 to 6. Table 2 shows that participants with NAFLD consumed more fatty fish, aquatic and sea food (all *P* < 0.05). No significant differences in NAFLD prevalence were found by groups of other fishes and meat consumption (*P* from 0.15 to 0.96) (Table 2).

**Table 2:** Description of fish and meat consumption by NAFLD on 1,862 participants of the Guangzhou Biobank Cohort Study

	Total (n = 1,862)	NAFLD		*P*
		Without (n = 1,169)	With (n = 693)	
**Fatty fish, n (%), servings/week, one serving = 50 g**				**0.04**
0	1,521 (81.69)	971 (83.06)	550 (79.37)	
1–2	116 (6.23)	74 (6.33)	42 (6.06)	
≥ 3	225 (12.08)	124 (10.61)	101 (14.57)	
**Other fishes, n (%), servings/week, one serving = 50 g**				**0.15**
0–3	393 (21.11)	248 (21.21)	145 (20.92)	
4–6	560 (30.08)	333 (28.49)	227 (32.76)	
7–10	644 (34.59)	424 (36.27)	220 (31.75)	
≥ 11	265 (14.23)	164 (14.03)	101 (14.57)	
**Aquatic and sea food, n (%), servings/week, one serving = 50 g**				**0.01**
0	1,382 (74.22)	873 (74.68)	509 (73.45)	
1–2	324 (17.40)	214 (18.31)	110 (15.87)	
≥ 3	156 (8.38)	82 (7.01)	74 (10.68)	
**Red meat, n (%), servings/week, one serving = 50 g**				**0.96**
0–3	318 (17.08)	201 (17.19)	117 (16.88)	
4–6	658 (35.34)	417 (35.67)	241 (34.78)	
7–10	596 (32.01)	369 (31.57)	227 (32.76)	
≥ 11	290 (15.57)	182 (15.57)	108 (15.58)	
**Poultry, n (%), servings/week, one serving = 50 g**				**0.64**
0–3	744 (39.96)	475 (40.63)	269 (38.82)	
4–6	670 (35.98)	424 (36.27)	246 (35.50)	
7–10	411 (22.07)	247 (21.13)	164 (23.67)	
≥ 11	37 (1.99)	23 (1.97)	14 (2.02)	
**Processed meat, n (%), servings/week, one serving = 50 g**				**0.58**
0	1,222 (65.63)	759 (64.93)	463 (66.81)	
1–2	569 (30.56)	367 (31.39)	202 (29.15)	
≥ 3	71 (3.81)	43 (3.68)	28 (4.04)	

NAFLD, non-alcoholic fatty liver disease

### Associations of fish and meat consumption with NAFLD

Table 3 shows that, after adjustment for age, sex, education, family income, occupation, smoking status, drinking status and physical activity, compared with those without fatty fish consumption, the ORs (95% CIs) for NAFLD was 1.05 (0.72, 1.54) and 1.40 (1.06, 1.86) in those with consumption of 1–2 servings/week and ≥ 3 servings/week, respectively (*P* for trend was 0.02, Model 2). After further adjustment for metabolic traits which did not significantly mediate the association of fatty fish with NAFLD (i.e., BMI, waist circumference, FPG, SBP, DBP, TG, TC, HDL-C and LDL-C) (Table 4), significant association between higher consumption of fatty fish and a higher risk of NAFLD remained. Compared with no consumption, the ORs (95% CIs) for NAFLD was 1.07 (0.64, 1.79) and 1.64 (1.12, 2.39) in consumption of 1–2 servings/week and ≥ 3 servings/week, respectively (*P* for trend was 0.01, Model 3). However, after similar adjustment, no significant association of other fishes, aquatic and sea food, red meat, poultry and processed meat intakes with NAFLD was found. The ORs (95% CIs) were 1.08 (0.70, 1.66), 1.41 (0.90, 2.21), 1.17 (0.75, 1.81), 1.02 (0.42, 2.50) and 0.85 (0.50, 1.45) in the highest, versus lowest consumption, respectively (Model 3).

**Table 3:** Association of fish and meat consumption with NAFLD in the Guangzhou Biobank Cohort Study

	OR (95% CI) for NAFLD			
	Crude Model	Model 1	Model 2	Model 3
**Fatty fish, servings/week, one serving = 50 g**				
0	1.00	1.00	1.00	1.00
1–2	1.14 (0.79, 1.65)	1.10 (0.76, 1.60)	1.05 (0.72, 1.54)	1.07 (0.64, 1.79)
≥ 3	1.39 (1.06, 1.84) ^*^	1.36 (1.03, 1.79) ^*^	1.40 (1.06, 1.86) ^*^	1.64 (1.12, 2.39) ^*^
Pseudo R-square	0.0022	0.0105	0.0231	0.3532
* P* for trend	0.02	0.03	0.02	0.01
**Other fish, servings/week, one serving = 50 g**				
0–3	1.00	1.00	1.00	1.00
4–6	1.19 (0.92, 1.54)	1.14 (0.88, 1.47)	1.09 (0.84, 1.42)	1.16 (0.81, 1.64)
7–10	0.90 (0.70, 1.16)	0.84 (0.65, 1.08)	0.83 (0.64, 1.08)	0.73 (0.52, 1.04)
≥ 11	1.01 (0.74, 1.38)	0.97 (0.71, 1.33)	0.95 (0.69, 1.30)	1.08 (0.70, 1.66)
Pseudo R-square	0.0022	0.0113	0.0233	0.3545
* P* for trend	0.37	0.21	0.19	0.29
**Aquatic and sea food, servings/week, one serving = 50 g**				
0	1.00	1.00	1.00	1.00
1–2	0.90 (0.70, 1.15)	0.89 (0.70, 1.14)	0.86 (0.67, 1.11)	1.21 (0.88, 1.68)
≥ 3	1.62 (1.17, 2.24) ^**^	1.52 (1.09, 2.10) ^*^	1.49 (1.06, 2.08) ^*^	1.41 (0.90, 2.21)
Pseudo R-square	0.0039	0.0118	0.0243	0.3505
* P* for trend	0.05	0.11	0.19	0.08
**Red meat, servings/week, one serving = 50 g**				
0–3	1.00	1.00	1.00	1.00
4–6	1.02 (0.78, 1.34)	1.00 (0.76, 1.31)	0.97 (0.73, 1.27)	1.02 (0.71, 1.47)
7–10	1.09 (0.83, 1.44)	1.05 (0.80, 1.38)	1.03 (0.77, 1.36)	1.07 (0.74, 1.55)
≥ 11	1.04 (0.76, 1.43)	0.99 (0.72, 1.37)	1.02 (0.73, 1.42)	1.17 (0.75, 1.81)
Pseudo R-square	0.0002	0.0088	0.0211	0.3500
* P* for trend	0.63	0.89	0.75	0.47
**Poultry, servings/week, one serving = 50 g**				
0–3	1.00	1.00	1.00	1.00
4–6	1.06 (0.86, 1.30)	1.02 (0.83, 1.26)	0.99 (0.80, 1.23)	0.94 (0.70, 1.25)
7–10	1.17 (0.92, 1.48)	1.09 (0.85, 1.39)	1.10 (0.86, 1.41)	0.95 (0.69, 1.33)
≥ 11	1.05 (0.55, 2.02)	0.97 (0.51, 1.88)	0.96 (0.49, 1.90)	1.02 (0.42, 2.50)
Pseudo R-square	0.0006	0.0089	0.0209	0.3498
* P* for trend	0.25	0.58	0.57	0.79
**Processed meat, servings/week, one serving = 50 g**				
0	1.00	1.00	1.00	1.00
1–2	0.94 (0.77, 1.14)	0.96 (0.78, 1.17)	0.91 (0.74, 1.12)	0.88 (0.70, 1.11)
≥ 3	1.12 (0.70, 1.78)	1.11 (0.70, 1.78)	1.11 (0.68, 1.82)	0.85 (0.50, 1.45)
Pseudo R-square	0.0003	0.0088	0.0215	0.1072
* P* for trend	0.74	0.89	0.59	0.25

NAFLD, non-alcoholic fatty liver disease

Model 1, adjusted for age and sex

Model 2, additionally adjusted for education, family income, occupation, smoking status, drinking status and physical activity

Model 3, additionally adjusted for BMI, waist circumference, fasting plasma glucose, systolic blood pressure, diastolic blood pressure, triglycerides, total cholesterol, high-density lipoprotein cholesterol or low-density lipoprotein cholesterol

*, *P* < 0.05; **, *P* < 0.01

**Table 4:** Mediation and suppression effects of metabolic traits on the associations of fish and meat consumption with NAFLD

Mediator or suppressor	Coefficients (95% CI) for indirect effect					
	Fatty fish	Other fish	Aquatic and sea food	Red meat	Poultry	Processed meat
BMI, kg/m^2^	0.025 (-0.077, 0.127)	-0.045 (-0.121, 0.031)	0.050 (-0.066, 0.166)	-0.031 (-0.096, 0.034)	0.034 (-0.049, 0.117)	0.158 (0.029, 0.287) ^*^
WC, cm	0.001 (-0.115, 0.116)	-0.009 (-0.084, 0.065)	0.049 (-0.088, 0.185)	-0.052 (-0.136, 0.032)	0.047 (-0.050, 0.143)	0.203 (0.058, 0.348) ^**^
Log-FPG, mmol/L	0.019 (-0.011, 0.048)	-0.009 (-0.029, 0.012)	0.001 (-0.035, 0.037)	-0.010 (-0.032, 0.012)	0.002 (-0.021, 0.025)	0.054 (0.017, 0.091) ^**^
SBP, mmHg	0.022 (-0.004, 0.047)	0.007 (-0.012, 0.026)	-0.049 (-0.077, -0.020) ^**^	-0.032 (-0.052, -0.013) ^**^	-0.008 (-0.031, 0.014)	0.021 (-0.011, 0.052)
DBP, mmHg	0.029 (-0.003, 0.061)	0.005 (-0.014, 0.024)	-0.013 (-0.043, 0.017)	-0.016 (-0.038, 0.007)	-0.003 (-0.031, 0.025)	0.032 (-0.009, 0.073)
Log-TG, mmol/L	-0.007 (-0.054, 0.040)	0.006 (-0.027, 0.040)	-0.019 (-0.074, 0.035)	-0.022 (-0.054, 0.009)	0.018 (-0.019, 0.055)	0.068 (0.005, 0.131) ^*^
TC, mmol/L	0.001 (-0.003, 0.003)	0.001 (-0.003, 0.003)	0.001 (-0.008, 0.009)	-0.001 (-0.003, 0.002)	-0.001 (-0.003, 0.003)	0.002 (-0.010, 0.013)
HDL-C, mmol/L	-0.029 (-0.070, 0.012)	-0.009 (-0.038, 0.021)	-0.060 (-0.108, -0.012) ^*^	0.001 (-0.027, 0.027)	0.004 (-0.028, 0.035)	0.007 (-0.036, 0.050)
LDL-C, mmol/L	0.002 (-0.00, 0.006)	-0.001 (-0.003, 0.003)	0.002 (-0.004, 0.009)	-0.001 (-0.003, 0.003)	-0.002 (-0.007, 0.003)	-0.001 (-0.005, 0.005)

NAFLD, non-alcoholic fatty liver disease; BMI, body mass index; WC, waist circumference; Log-FPG, log-transformed fasting plasma glucose; SBP, systolic blood pressure; DBP, diastolic blood pressure; Log-TG, log-transformed triglycerides; TC, total cholesterol; HDL-C, high-density lipoprotein cholesterol; LDL-C, low-density lipoprotein cholesterol. *, *P* < 0.05. **, *P* < 0.01

Table 4 shows significant negative indirect effects of aquatic and sea food on NAFLD through SBP and HDL-C, with β for indirect effect (95% CI) being − 0.049 (-0.077, -0.020) and − 0.060 (-0.108, -0.012), respectively (all *P* < 0.05). The similar suppression effect of SBP was also found on the association between red meat and NAFLD (-0.032 (-0.052, -0.013); *P* < 0.01). Significant positive indirect effects of processed meat on NAFLD mediated by BMI, waist circumference, log-FPG and log-TG were found (0.158 (0.029, 0.287); 0.203 (0.058, 0.348); 0.054 (0.017, 0.091) and 0.068 (0.005, 0.131), respectively; all *P* < 0.05). No significant indirect effect of fatty fish, other fish and poultry on NAFLD was found.

## Discussion

In this population-based cross-sectional study of 1,862 middle-aged and older adults, we found that compared with no consumption (0 serving/week), consumption of fatty fish of ≥ 3 servings/week was associated with higher odds of NAFLD by 64%. However, no significant association of other fishes, aquatic and sea food, red meat, poultry and processed meat with NAFLD was observed. Our findings suggest reducing fatty fish consumption may decrease the risk of NAFLD, and also underline the importance of unravelling different dietary compositions in preventing NAFLD.

The associations of fish and meat consumption with NAFLD were reported in a few studies [22–25]. Our findings were generally consistent with the previous studies and added to the literature by showing positive association of fatty fish with NAFLD, which were not reported before [22–25]. For example, a cross-sectional study of 1,594 Chinese reported no association of poultry and processed meat consumption with the presence of NAFLD, but showed that per 50 g/day higher red meat consumption was associated with 14% higher odds of NAFLD [22]. A cohort study of 1,340 Iranian with low meat consumption also found no association of processed meat and chicken consumption with NAFLD, but higher red meat consumption (about 34 g/day), versus low consumption (about 4 g/day), was associated with higher risk of NAFLD during a follow-up of 6 years (OR (95% CI): 1.59 (1.06, 2.38 )) [23]. A nested case-control study on ethnically diverse population also found that higher red meat and poultry consumption were related to the presence of NAFLD [24]. In addition, the positive association between red meat consumption and NAFLD was further supported by a recent meta-analysis [25]. One of the explanations for the discrepancies between findings from these studies and ours may be due to different cooking method (i.e., mostly steaming in our study sample). The detrimental effect of red meat may be partly attributable to heterocyclic aromatic amines (HAAs) informed during the unhealthy cooking methods, such as frying and barbecuing [24]. HAAs have been shown to increase oxidative stress, which plays a key role in the progression of NAFLD [24]. Another explanation may be due to the different intake of poultry in the population level. The median level of poultry consumption in the West was about 37.8 g/day [24], which was higher than that in our sample (about 28.6 g/day).

The association of fatty fish with NAFLD was also inconclusive in previous studies. A cohort study of 43,655 middle-aged and older adults in South Korea showed that higher fatty fish consumption was associated with lower risk of NAFLD in women during a follow-up of 5 years (highest versus lowest quartile of consumption, HR (95% CI): 0.84 (0.78, 0.90), *P* for trend < 0.5) [26]. The discrepancy between this study and ours may be due to the different method for NAFLD assessment. Specifically, NAFLD was diagnosed by fatty liver index (i.e., calculated by parameter including BMI, waist circumference, triglycerides and γ-glutamyl transferase) in the Korean study whilst by abdominal ultrasonography in our study, and the diagnosis based on ultrasound is more accurate. Fish is regarded as a source of n-3 PUFAs with potential protective effects for health [37], but fatty fish also contain fat-soluble persistent organic pollutants (POPs) [38], which have been linked to a higher risk of type 2 diabetes [39]. Thus the potentially beneficial effects of n-3 PUFAs may be counteracted by elevated level of POPs [38]. Note that a previous experimental study showed that POPs in fatty fish increased the risk of insulin resistance syndrome including metabolic syndrome and type 2 diabetes [40]. In addition, a meta-analysis of cohort studies found a positive association between fish consumption and risk of type 2 diabetes in American and European who prefer fatty fish, but such association was not evident in Asian [41]. As NAFLD shares similar pathogenesis with type 2 diabetes [22], our results were to some extent consistent with the findings of the meta-analysis.

The suppression effects of SBP and HDL-C on the associations of red meat, and aquatic and sea food with NAFLD might be due to the negative associations of red meat, and aquatic and sea food with SBP, as well as the positive association of aquatic and sea food with HDL-C in our samples. Our results were generally consistent with a study in Australians, showing that replacing energy intake from carbohydrate with lean red meat (180 or 250 g raw weight) was associated with 4 mmHg reduction in SBP [42]. Another study in Chinese also reported a negative association of dietary fat taken from seafood with hypertension risk [43]. As hypertension and lower HDL-C have been reported to be risk factors of NAFLD [44], red meat, and aquatic and sea food thus showed potential negative indirect associations with NAFLD through SBP and HDL-C.

There were several limitations in our study. First, the casual inference of fatty fish, aquatic and sea food with NAFLD could not be confirmed in this cross-sectional study. Second, recall error were inevitable for self-report diet, although we used a catalogue of pictures of individual food portions to explain specific portion sizes, which might minimize the recall error. The existence of recall error might lead to underestimated associations. Third, we measured diet during the past seven days rather than assessed the long-time eating habits. However, as diet habit in older Chinese is relatively stable, this measurement method might not be a major concern. Fourth, residual confounding cannot be completely ruled out, although we adjusted for multiple potential confounders. Fifth, while liver biopsy is the gold standard for diagnosing NAFLD, we opted for ultrasonography due to its non-invasive nature, making it more apt for general population studies. It is worth noting that ultrasound exhibits robust sensitivity and specificity in detecting moderate to severe steatosis, even though it might miss milder cases. The strengths of our study included the use of standardized data collection procedure and comprehensive adjustment for potential confounders.

## Conclusion

In conclusion, we found positive association of fatty fish with NAFLD in middle-aged and older Chinese. Our findings suggest that reduction of fatty fish consumption might be beneficial in prevention of NAFLD in older adults.

### Electronic supplementary material

Below is the link to the electronic supplementary material.

Supplementary Material 1

## Data Availability

Due to privacy or ethical restrictions, the data that support the findings will be made available on requests from the Guangzhou Biobank Cohort Study Data Access Committee (kh.ukh@atadscbg). The datasets used and/or analyzed during the current study are available from the corresponding author on reasonable request.

## Abbreviations

NAFLD: Non-alcoholic fatty liver disease
OR: Odds ratio
CI: Confidence interval
n-3 PUFAs: Omega-3 polyunsaturated fatty acids
IR: Insulin resistance
GBCS: Guangzhou Biobank Cohort Study
FFQ: Food-frequency questionnaire
FA: Fatty acid
EPA: Eicosapentaenoic acid
DHA: Docosahexaenoic acid
FPG: Fasting plasma glucose
TC: Total cholesterol
TG: Triglycerides
HDL-C: High-density lipoprotein cholesterol
LDL-C: Low-density lipoprotein cholesterol
BMI: Body mass index
SD: Standard deviation
IQR: Interquartile range
SBP: Systolic blood pressure
DBP: Diastolic blood pressure
HAAs: Heterocyclic aromatic amines
POPs: Persistent organic pollutants
CNY: Chinese Yuan (US$1 = 7 CNY)
