# Supplementary material for: Association of fish and meat consumption with non-alcoholic fatty liver disease: Guangzhou Biobank Cohort Study
Source: BMC Public Health. 2023 Dec 6;23:2433. doi: 10.1186/s12889-023-17398-6 (PMC10699064; doi:10.1186/s12889-023-17398-6)
Supplement: Supplementary file 1 — Supplementary Material 1 [file 12889_2023_17398_MOESM1_ESM.docx]

**Supplementary table 1 Characteristics of study sample by fatty fish consumption on 1,862 participants of the Guangzhou Biobank Cohort Study**

|  | Fatty fish consumption, servings per week (one serving = 50 g) | | | *P* |
| --- | --- | --- | --- | --- |
|  | 0 (n = 1,521) | 1-2 (n = 116) | ≥ 3 (n = 225) |  |
| Age (years), mean (SD) | 61.13 (6.54) | 60.11 (6.19) | 59.75 (6.19) | < 0.01 |
| Sex, n (%) |  |  |  | 0.06 |
| Men | 739 (48.59) | 62 (53.45) | 127 (56.44) |  |
| Women | 782 (51.41) | 54 (46.55) | 98 (43.56) |  |
| Education, n (%) |  |  |  | 0.001 |
| Primary or below | 561 (36.88) | 45 (38.79) | 58 (25.78) |  |
| Middle school | 788 (51.81) | 50 (43.10) | 141 (62.67) |  |
| College or above | 172 (11.31) | 21 (18.10) | 26 (11.56) |  |
| Family income (CNY/year), n (%) |  |  |  | 0.48 |
| < 10,000 | 18 (1.18) | 0 (0.00) | 5 (2.22) |  |
| 10,000-29,999 | 177 (11.64) | 11 (9.48) | 21 (9.33) |  |
| 30,000-49,999 | 435 (28.60) | 30 (25.86) | 61 (27.11) |  |
| ≥ 50,000 | 185 (12.16) | 19 (16.38) | 34 (15.11) |  |
| Don’t know | 706 (46.42) | 56 (48.28) | 104 (46.22) |  |
| Occupation, n (%) |  |  |  | 0.90 |
| Manual | 844 (55.49) | 62 (53.45) | 119 (52.89) |  |
| Non-manual | 386 (25.38) | 33 (28.45) | 62 (27.56) |  |
| Other | 291 (19.13) | 21 (18.10) | 44 (19.56) |  |
| Drinking status, n (%) |  |  |  | 0.42 |
| Never | 425 (27.94) | 26 (22.41) | 60 (26.67) |  |
| Ever | 1,096 (72.06) | 90 (77.59) | 165 (73.33) |  |
| Smoking status, n (%) |  |  |  | 0.28 |
| Never | 1,058 (69.56) | 81 (69.83) | 150 (66.67) |  |
| Former | 218 (14.33) | 12 (10.34) | 28 (12.44) |  |
| Current | 245 (16.11) | 23 (19.83) | 47 (20.89) |  |
| Physical activity, n (%) |  |  |  | 0.23 |
| Low | 12 (0.79) | 1 (0.86) | 0 (0.00) |  |
| Moderate | 393 (25.84) | 22 (18.97) | 64 (28.44) |  |
| Active | 1,116 (73.37) | 93 (80.17) | 161 (71.56) |  |
| BMI (kg/m^2^), mean (SD) | 23.84 (3.46) | 23.94 (3.13) | 23.86 (3.56) | 0.96 |
| Waist circumference (cm), mean (SD) | 83.37 (9.30) | 83.50 (8.82) | 83.23 (9.84) | 0.97 |
| SBP (mmHg), mean (SD) | 130.55 (19.60) | 128.23 (17.51) | 132.04 (20.84) | 0.23 |
| DBP (mmHg), mean (SD) | 73.19 (10.25) | 73.03 (8.88) | 75.05 (10.91) | 0.08 |
| FPG (mmol/L), median (IQR) | 5.08 (4.72, 5.54) | 5.07 (4.72, 5.60) | 5.12 (4.71, 5.72) | 0.59 |
| TC (mmol/L), mean (SD) | 5.67 (1.15) | 5.75 (1.07) | 5.64 (0.99) | 0.67 |
| TG (mmol/L), median (IQR) | 1.45 (1.05, 2.12) | 1.28 (0.99, 1.96) | 1.47 (1.00, 2.15) | 0.40 |
| HDL-C (mmol/L), mean (SD) | 1.31 (0.30) | 1.35 (0.30) | 1.32 (0.29) | 0.49 |
| LDL-C (mmol/L), mean (SD) | 3.37 (0.89) | 3.39 (0.85) | 3.42 (0.93) | 0.74 |

CNY, Chinese Yuan (US$1 = 7CNY); SD, standard deviation; IQR, interquartile range; BMI, body mass index; SBP, systolic blood pressure; DBP, diastolic blood pressure; FPG, fasting plasma-glucose; TC, total cholesterol; TG, triglycerides; HDL-C, high-density lipoprotein cholesterol; LDL-C, low-density lipoprotein cholesterol.

**Supplementary table 2 Characteristics of study sample by other fish consumption on 1,862 participants of the Guangzhou Biobank Cohort Study**

|  | Other fish consumption, servings per week (one serving = 50 g) | | | | *P* |
| --- | --- | --- | --- | --- | --- |
|  | 0-3 (n = 393) | 4-6 (n = 560) | 7-10 (n = 644) | ≥ 11 (n = 265) |  |
| Age (years), mean (SD) | 60.91 (6.36) | 61.13 (6.69) | 61.03 (6.56) | 60.07 (6.04) | 0.15 |
| Sex, n (%) |  |  |  |  | < 0.001 |
| Men | 155 (39.44) | 287 (51.25) | 357 (55.43) | 129 (48.68) |  |
| Women | 238 (60.56) | 273 (48.75) | 287 (44.57) | 136 (51.32) |  |
| Education, n (%) |  |  |  |  | 0.29 |
| Primary or below | 149 (37.91) | 208 (37.14) | 211 (32.76) | 96 (36.23) |  |
| Middle school | 207 (52.67) | 278 (48.64) | 353 (54.81) | 141 (53.21) |  |
| College or above | 37 (9.41) | 74 (13.21) | 80 (12.42) | 28 (10.27) |  |
| Family income (CNY/year), n (%) |  |  |  |  | 0.07 |
| < 10,000 | 5 (1.27) | 7 (1.25) | 4 (0.62) | 7 (2.64) |  |
| 10,000-29,999 | 58 (14.76) | 64 (11.43) | 61 (9.47) | 26 (9.81) |  |
| 30,000-49,999 | 105 (26.72) | 142 (25.36) | 205 (31.83) | 74 (27.92) |  |
| ≥ 50,000 | 45 (11.45) | 73 (13.04) | 86 (13.35) | 34 (12.83) |  |
| Don’t know | 180 (45.80) | 274 (48.93) | 288 (44.72) | 124 (46.79) |  |
| Occupation, n (%) |  |  |  |  | 0.03 |
| Manual | 231 (58.78) | 285 (50.89) | 367 (56.99) | 142 (53.58) |  |
| Non-manual | 80 (20.36) | 170 (30.36) | 162 (25.16) | 69 (26.04) |  |
| Other | 82 (20.87) | 105 (18.75) | 115 (17.86) | 54 (20.38) |  |
| Drinking status, n (%) |  |  |  |  | 0.24 |
| Never | 122 (31.04) | 140 (25.00) | 176 (27.33) | 73 (27.55) |  |
| Ever | 271 (68.96) | 420 (75.00) | 468 (72.67) | 192 (72.45) |  |
| Smoking status, n (%) |  |  |  |  | 0.01 |
| Never | 300 (76.34) | 388 (69.29) | 419 (65.06) | 182 (68.68) |  |
| Former | 43 (10.94) | 84 (15.00) | 94 (14.60) | 37 (13.96) |  |
| Current | 50 (12.72) | 88 (15.71) | 131 (20.34) | 46 (17.36) |  |
| Physical activity, n (%) |  |  |  |  | < 0.01 |
| Low | 5 (1.27) | 3 (0.54) | 5 (0.78) | 0 (0.00) |  |
| Moderate | 112 (28.50) | 160 (28.57) | 160 (24.84) | 47 (17.74) |  |
| Active | 276 (70.23) | 397 (70.89) | 479 (74.38) | 218 (82.26) |  |
| BMI (kg/m^2^), mean (SD) | 23.90 (3.44) | 24.03 (3.39) | 23.78 (3.39) | 23.55 (3.75) | 0.28 |
| Waist circumference (cm), mean (SD) | 82.94 (9.13) | 83.68 (9.28) | 83.63 (9.33) | 82.65 (9.74) | 0.32 |
| SBP (mmHg), mean (SD) | 130.46 (20.51) | 130.70 (19.24) | 130.57 (19.73) | 130.54 (18.98) | 1.00 |
| DBP (mmHg), mean (SD) | 73.41 (10.30) | 72.88 (9.97) | 73.79 (10.46) | 73.56 (10.36) | 0.49 |
| FPG (mmol/L), median (IQR) | 5.09 (4.71, 5.59) | 5.10 (4.73, 5.59) | 5.08 (4.74, 5.56) | 5.06 (4.66, 5.51) | 0.52 |
| TC (mmol/L), mean (SD) | 5.65 (1.04) | 5.75 (1.24) | 5.60 (1.05) | 5.69 (1.21) | 0.74 |
| TG (mmol/L), median (IQR) | 1.44 (1.08, 2.04) | 1.43 (1.00, 2.13) | 1.46 (1.05, 2.10) | 1.48 (1.02, 2.16) | 0.94 |
| HDL-C (mmol/L), mean (SD) | 1.32 (0.27) | 1.31 (0.31) | 1.30 (0.31) | 1.33 (0.30) | 0.35 |
| LDL -C(mmol/L), mean (SD) | 3.38 (0.90) | 3.42 (0.92) | 3.35 (0.83) | 3.37 (0.97) | 0.94 |

CNY, Chinese Yuan (US$1 = 7CNY); SD, standard deviation; IQR, interquartile range; BMI, body mass index; SBP, systolic blood pressure; DBP, diastolic blood pressure; FPG, fasting plasma-glucose; TC, total cholesterol; TG, triglycerides; HDL-C, high-density lipoprotein cholesterol; LDL-C, low-density lipoprotein cholesterol.

**Supplementary table 3 Characteristics of study sample by aquatic and sea food consumption on 1,862 participants of the Guangzhou Biobank Cohort Study**

|  | Aquatic and sea food consumption, servings per week (one serving = 50 g) | | | *P* |
| --- | --- | --- | --- | --- |
|  | 0 (n = 1,382) | 1-2 (n = 324) | ≥ 3 (n = 156) |  |
| Age (years), mean (SD) | 61.04 (6.55) | 60.56 (6.10) | 60.35 (6.72) | 0.27 |
| Sex, n (%) |  |  |  | < 0.001 |
| Men | 671 (48.55) | 155 (47.84) | 102 (65.38) |  |
| Women | 711 (51.45) | 169 (52.16) | 54 (34.62) |  |
| Education, n (%) |  |  |  | 0.02 |
| Primary or below | 514 (37.19) | 102 (31.48) | 48 (30.77) |  |
| Middle school | 723 (52.32) | 175 (54.01) | 81 (51.92) |  |
| College or above | 145 (10.49) | 47 (14.51) | 27 (17.31) |  |
| Family income (CNY/year), n (%) |  |  |  | 0.07 |
| < 10,000 | 14 (1.01) | 3 (0.93) | 6 (3.85) |  |
| 10,000-29,999 | 159 (11.51) | 30 (9.26) | 20 (12.82) |  |
| 30,000-49,999 | 387 (28.00) | 95 (29.32) | 44 (28.21) |  |
| ≥ 50,000 | 172 (12.45) | 41 (12.65) | 25 (16.03) |  |
| Don’t know | 650 (47.03) | 155 (47.84) | 61 (39.10) |  |
| Occupation, n (%) |  |  |  | 0.86 |
| Manual | 764 (55.28) | 175 (54.01) | 86 (55.13) |  |
| Non-manual | 352 (25.47) | 91 (28.09) | 38 (24.36) |  |
| Other | 266 (19.25) | 58 (17.90) | 32 (20.51) |  |
| Drinking status, n (%) |  |  |  | < 0.001 |
| Never | 419 (30.32) | 64 (19.75) | 28 (17.95) |  |
| Ever | 963 (69.68) | 260 (80.25) | 128 (82.05) |  |
| Smoking status, n (%) |  |  |  | 0.03 |
| Never | 968 (70.04) | 231 (71.30) | 90 (57.69) |  |
| Former | 187 (13.53) | 42 (12.96) | 29 (18.59) |  |
| Current | 227 (16.43) | 51 (15.74) | 37 (23.72) |  |
| Physical activity, n (%) |  |  |  | 0.02 |
| Low | 10 (0.72) | 2 (0.62) | 1 (0.64) |  |
| Moderate | 331 (23.95) | 92 (28.40) | 56 (35.90) |  |
| Active | 1,041 (75.33) | 230 (70.99) | 99 (63.46) |  |
| BMI (kg/m^2^), mean (SD) | 23.87 (3.45) | 23.51 (3.22) | 24.34 (3.86) | 0.03 |
| Waist circumference (cm), mean (SD) | 83.42 (9.50) | 81.93 (8.27) | 85.79 (9.46) | < 0.001 |
| SBP (mmHg), mean (SD) | 131.56 (19.92) | 127.95 (18.66) | 127.39 (18.27) | < 0.01 |
| DBP (mmHg), mean (SD) | 73.50 (10.38) | 72.67 (9.24) | 74.06 (11.18) | 0.27 |
| FPG (mmol/L), median (IQR) | 5.09 (4.73, 5.57) | 5.03 (4.71, 5.46) | 5.15 (4.68, 5.68) | 0.24 |
| TC (mmol/L), mean (SD) | 5.65 (1.16) | 5.70 (1.02) | 5.75 (1.13) | 0.45 |
| TG (mmol/L), median (IQR) | 1.45 (1.05, 2.10) | 1.49 (1.01, 2.11) | 1.31 (1.06, 2.19) | 0.63 |
| HDL-C (mmol/L), mean (SD) | 1.30 (0.30) | 1.36 (0.30) | 1.31 (0.29) | 0.01 |
| LDL-C (mmol/L), mean (SD) | 3.37 (0.90) | 3.38 (0.88) | 3.48 (0.90) | 0.35 |

CNY, Chinese Yuan (US$1 = 7CNY); SD, standard deviation; IQR, interquartile range; BMI, body mass index; SBP, systolic blood pressure; DBP, diastolic blood pressure; FPG, fasting plasma-glucose; TC, total cholesterol; TG, triglycerides; HDL-C, high-density lipoprotein cholesterol; LDL-C, low-density lipoprotein cholesterol.

**Supplementary table 4 Characteristics of study sample by red meat consumption on 1,862 participants of the Guangzhou Biobank Cohort Study**

|  | Red meat consumption, servings per week (one serving = 50 g) | | | | *P* |
| --- | --- | --- | --- | --- | --- |
|  | 0-3 (n = 318) | 4-6 (n = 658) | 7-10 (n = 596) | ≥ 11 (n = 290) |  |
| Age (years), mean (SD) | 61.37 (6.38) | 60.69 (6.37) | 61.11 (6.62) | 60.43 (6.57) | 0.22 |
| Sex, n (%) |  |  |  |  | 0.01 |
| Men | 139 (43.71) | 314 (47.72) | 320 (53.69) | 155 (53.45) |  |
| Women | 179 (56.29) | 344 (52.28) | 276 (46.31) | 135 (46.55) |  |
| Education, n (%) |  |  |  |  | 0.56 |
| Primary or below | 123 (38.68) | 232 (35.26) | 210 (35.23) | 99 (34.14) |  |
| Middle school | 152 (47.80) | 349 (53.04) | 324 (54.36) | 154 (53.10) |  |
| College or above | 43 (13.52) | 77 (11.70) | 62 (10.40) | 37 (12.76) |  |
| Family income (CNY/year), n (%) |  |  |  |  | 0.13 |
| < 10,000 | 3 (0.94) | 10 (1.52) | 9 (1.51) | 1 (0.34) |  |
| 10,000-29,999 | 38 (11.95) | 77 (11.70) | 54 (9.06) | 40 (13.79) |  |
| 30,000-49,999 | 96 (30.19) | 202 (30.70) | 163 (27.35) | 65 (22.41) |  |
| ≥ 50,000 | 40 (12.58) | 86 (13.07) | 72 (12.08) | 40 (13.79) |  |
| Don’t know | 141 (44.34) | 283 (43.01) | 298 (50.00) | 144 (49.66) |  |
| Occupation, n (%) |  |  |  |  | 0.90 |
| Manual | 172 (54.09) | 357 (54.26) | 333 (55.87) | 163 (56.21) |  |
| Non-manual | 87 (27.36) | 167 (25.38) | 157 (26.34) | 70 (24.14) |  |
| Other | 59 (18.55) | 134 (20.36) | 106 (17.79) | 57 (19.66) |  |
| Drinking status, n (%) |  |  |  |  | 0.76 |
| Never | 85 (26.73) | 177 (26.90) | 173 (29.03) | 76 (26.21) |  |
| Ever | 233 (73.27) | 481 (73.10) | 423 (70.97) | 214 (73.79) |  |
| Smoking status, n (%) |  |  |  |  | 0.01 |
| Never | 238 (74.84) | 472 (71.73) | 397 (66.61) | 182 (62.76) |  |
| Former | 43 (13.52) | 85 (12.92) | 87 (14.60) | 43 (14.83) |  |
| Current | 37 (11.64) | 101 (15.35) | 112 (18.79) | 65 (22.41) |  |
| Physical activity, n (%) |  |  |  |  | < 0.001 |
| Low | 2 (0.63) | 8 (1.22) | 2 (0.34) | 1 (0.34) |  |
| Moderate | 97 (30.50) | 192 (29.18) | 147 (24.66) | 43 (14.83) |  |
| Active | 219 (68.87) | 458 (69.60) | 447 (75.00) | 246 (84.83) |  |
| BMI (kg/m^2^), mean (SD) | 23.90 (3.41) | 23.94 (3.32) | 23.78 (3.57) | 23.72 (3.56) | 0.76 |
| Waist circumference (cm), mean (SD) | 83.46 (9.66) | 83.55 (8.58) | 83.30 (9.59) | 82.96 (10.09) | 0.80 |
| SBP (mmHg), mean (SD) | 133.72 (20.58) | 130.15 (19.53) | 130.54 (19.01) | 128.19 (19.74) | 0.01 |
| DBP (mmHg), mean (SD) | 73.90 (10.56) | 73.26 (9.98) | 73.43 (10.23) | 73.13 (10.67) | 0.78 |
| FPG (mmol/L), median (IQR) | 5.12 (4.74, 5.69) | 5.06 (4.7, 5.58) | 5.09 (4.74, 5.52) | 5.07 (4.69, 5.51) | 0.43 |
| TC (mmol/L), mean (SD) | 5.77 (1.09) | 5.69 (1.18) | 5.58 (1.10) | 5.70 (1.12) | 0.09 |
| TG (mmol/L), median (IQR) | 1.55 (1.10, 2.14) | 1.46 (1.02, 2.19) | 1.43 (1.04, 2.05) | 1.37 (1.02, 2.05) | 0.24 |
| HDL-C (mmol/L), mean (SD) | 1.32 (0.29) | 1.32 (0.31) | 1.30 (0.30) | 1.31 (0.28) | 0.57 |
| LDL-C (mmol/L), mean (SD) | 3.40 (0.92) | 3.40 (0.93) | 3.34 (0.84) | 3.39 (0.90) | 0.67 |

CNY, Chinese Yuan (US$1 = 7CNY); SD, standard deviation; IQR, interquartile range; BMI, body mass index; SBP, systolic blood pressure; DBP, diastolic blood pressure; FPG, fasting plasma-glucose; TC, total cholesterol; TG, triglycerides; HDL-C, high-density lipoprotein cholesterol; LDL-C, low-density lipoprotein cholesterol.

**Supplementary table 5 Characteristics of study sample by poultry consumption on 1,862 participants of the Guangzhou Biobank Cohort Study**

|  | Poultry consumption, servings per week (one serving = 50 g) | | | | *P* |
| --- | --- | --- | --- | --- | --- |
|  | 0-3 (n = 744) | 4-6 (n = 670) | 7-10 (n = 411) | ≥ 11 (n = 37) |  |
| Age (years), mean (SD) | 61.23 (6.62) | 60.65 (6.38) | 60.66 (6.42) | 61.41 (6.37) | 0.28 |
| Sex, n (%) |  |  |  |  | < 0.001 |
| Men | 322 (43.28) | 340 (50.75) | 243 (59.12) | 23 (62.16) |  |
| Women | 422 (56.72) | 330 (49.25) | 168 (40.88) | 14 (37.84) |  |
| Education, n (%) |  |  |  |  | 0.02 |
| Primary or below | 295 (39.65) | 226 (33.73) | 125 (30.41) | 18 (48.65) |  |
| Middle school | 361 (48.52) | 367 (54.78) | 234 (56.93) | 17 (45.95) |  |
| College or above | 88 (11.83) | 77 (11.49) | 52 (12.65) | 2 (5.41) |  |
| Family income (CNY/year), n (%) |  |  |  |  | 0.39 |
| < 10,000 | 8 (1.08) | 10 (1.49) | 4 (0.97) | 1 (2.70) |  |
| 10,000-29,999 | 89 (11.96) | 68 (10.15) | 45 (10.95) | 7 (18.92) |  |
| 30,000-49,999 | 193 (25.94) | 198 (29.55) | 128 (31.14) | 7 (18.92) |  |
| ≥ 50,000 | 88 (11.83) | 95 (14.18) | 49 (11.92) | 6 (16.22) |  |
| Don’t know | 366 (49.19) | 299 (44.63) | 185 (45.01) | 16 (43.24) |  |
| Occupation, n (%) |  |  |  |  | 0.30 |
| Manual | 429 (57.66) | 360 (53.73) | 214 (52.07) | 22 (59.46) |  |
| Non-manual | 181 (24.33) | 177 (26.42) | 111 (27.01) | 12 (32.43) |  |
| Other | 134 (18.01) | 133 (19.85) | 86 (20.92) | 3 (8.11) |  |
| Drinking status, n (%) |  |  |  |  | 0.11 |
| Never | 224 (30.11) | 179 (26.72) | 97 (23.60) | 11 (29.73) |  |
| Ever | 520 (69.89) | 491 (73.28) | 314 (76.40) | 26 (70.27) |  |
| Smoking status, n (%) |  |  |  |  | < 0.001 |
| Never | 555 (74.60) | 465 (69.40) | 248 (60.34) | 21 (56.76) |  |
| Former | 85 (11.42) | 95 (14.18) | 71 (17.27) | 7 (18.92) |  |
| Current | 104 (13.98) | 110 (16.42) | 92 (22.38) | 9 (24.32) |  |
| Physical activity, n (%) |  |  |  |  | 0.09 |
| Low | 5 (0.67) | 5 (0.75) | 3 (0.73) | 0 (0.00) |  |
| Moderate | 181 (24.33) | 198 (29.55) | 95 (23.11) | 5 (13.51) |  |
| Active | 558 (75.00) | 467 (69.70) | 313 (76.16) | 32 (86.49) |  |
| BMI (kg/m^2^), mean (SD) | 23.86 (3.46) | 23.77 (3.54) | 23.97 (3.29) | 23.67 (3.59) | 0.83 |
| Waist circumference (cm), mean (SD) | 83.12 (9.70) | 83.14 (9.06) | 84.07 (9.09) | 84.30 (9.38) | 0.31 |
| SBP (mmHg), mean (SD) | 131.27 (19.65) | 129.94 (19.66) | 130.61 (19.67) | 128.27 (18.68) | 0.54 |
| DBP (mmHg), mean (SD) | 73.12 (10.41) | 73.52 (10.04) | 73.82 (10.29) | 72.41 (11.26) | 0.64 |
| FPG (mmol/L), median (IQR) | 5.09 (4.73, 5.59) | 5.04 (4.69, 5.52) | 5.12 (4.75, 5.60) | 5.23 (4.82, 5.69) | 0.25 |
| TC (mmol/L), mean (SD) | 5.75 (1.17) | 5.62 (1.10) | 5.60 (1.11) | 5.73 (0.92) | 0.07 |
| TG (mmol/L), median (IQR) | 1.46 (1.01, 2.05) | 1.45 (1.04, 2.15) | 1.43 (1.02, 2.21) | 1.29 (0.99, 1.88) | 0.84 |
| HDL-C (mmol/L), mean (SD) | 1.33 (0.31) | 1.31 (0.30) | 1.31 (0.29) | 1.28 (0.28) | 0.54 |
| LDL-C (mmol/L), mean (SD) | 3.44 (0.92) | 3.35 (0.88) | 3.31 (0.88) | 3.42 (0.65) | 0.03 |

CNY, Chinese Yuan (US$1 = 7CNY); SD, standard deviation; IQR, interquartile range; BMI, body mass index; SBP, systolic blood pressure; DBP, diastolic blood pressure; FPG, fasting plasma-glucose; TC, total cholesterol; TG, triglycerides; HDL-C, high-density lipoprotein cholesterol; LDL-C, low-density lipoprotein cholesterol.

**Supplementary table 6 Characteristics of study sample by processed meat consumption on 1,862 participants of the Guangzhou Biobank Cohort Study**

|  | Processed meat consumption, servings per week (one serving = 50 g) | | | *P* |
| --- | --- | --- | --- | --- |
|  | 0 (n = 1,222) | 1-2 (n = 569) | ≥ 3 (n = 71) |  |
| Age (years), mean (SD) | 60.88 (6.62) | 60.82 (6.17) | 61.91 (6.78) | 0.40 |
| Sex, n (%) |  |  |  | 0.07 |
| Men | 629 (51.47) | 261 (45.87) | 38 (53.52) |  |
| Women | 593 (48.53) | 308 (54.13) | 33 (46.48) |  |
| Education, n (%) |  |  |  | 0.03 |
| Primary or below | 415 (33.96) | 213 (37.43) | 36 (50.70) |  |
| Middle school | 655 (53.60) | 293 (51.49) | 31 (43.66) |  |
| College or above | 152 (12.44) | 63 (11.07) | 4 (5.63) |  |
| Family income (CNY/year), n (%) |  |  |  | < 0.001 |
| < 10,000 | 8 (0.65) | 13 (2.28) | 2 (2.82) |  |
| 10,000-29,999 | 136 (11.13) | 56 (9.84) | 17 (23.94) |  |
| 30,000-49,999 | 332 (27.17) | 181 (31.81) | 13 (18.31) |  |
| ≥ 50,000 | 149 (12.19) | 80 (14.06) | 9 (12.68) |  |
| Don’t know | 597 (48.85) | 239 (42.00) | 30 (42.25) |  |
| Occupation, n (%) |  |  |  | 0.11 |
| Manual | 653 (53.44) | 324 (56.94) | 48 (67.61) |  |
| Non-manual | 321 (26.27) | 145 (25.48) | 15 (21.13) |  |
| Other | 248 (20.29) | 100 (17.57) | 8 (11.27) |  |
| Drinking status, n (%) |  |  |  | < 0.001 |
| Never | 375 (30.69) | 122 (21.44) | 14 (19.72) |  |
| Ever | 847 (69.31) | 447 (78.56) | 57 (80.28) |  |
| Smoking status, n (%) |  |  |  | 0.64 |
| Never | 849 (69.48) | 396 (69.60) | 44 (61.97) |  |
| Former | 168 (13.75) | 76 (13.36) | 14 (19.72) |  |
| Current | 205 (16.78) | 97 (17.05) | 13 (18.31) |  |
| Physical activity, n (%) |  |  |  | 0.05 |
| Low | 6 (0.49) | 7 (1.23) | 0 (0.00) |  |
| Moderate | 293 (23.98) | 167 (29.35) | 19 (26.76) |  |
| Active | 923 (75.53) | 395 (69.42) | 52 (73.24) |  |
| BMI (kg/m^2^), mean (SD) | 23.73 (3.44) | 24.00 (3.47) | 24.64 (3.47) | 0.04 |
| Waist circumference (cm), mean (SD) | 83.05 (9.35) | 83.60 (9.20) | 86.76 (9.68) | < 0.01 |
| SBP (mmHg), mean (SD) | 130.45 (19.94) | 130.38 (19.12) | 134.53 (18.17) | 0.22 |
| DBP (mmHg), mean (SD) | 73.39 (10.22) | 73.03 (10.22) | 76.68 (10.84) | 0.02 |
| FPG (mmol/L), median (IQR) | 5.08 (4.71, 5.52) | 5.07 (4.71, 5.60) | 5.38 (4.87, 5.96) | 0.04 |
| TC (mmol/L), mean (SD) | 5.62 (1.10) | 5.75 (1.19) | 5.92 (1.11) | 0.01 |
| TG (mmol/L), median (IQR) | 1.43 (1.02, 2.10) | 1.47 (1.07, 2.09) | 1.66 (1.22, 2.47) | 0.01 |
| HDL-C (mmol/L), mean (SD) | 1.31 (0.30) | 1.32 (0.30) | 1.28 (0.26) | 0.39 |
| LDL-C (mmol/L), mean (SD) | 3.38 (0.86) | 3.38 (0.97) | 3.34 (0.85) | 0.93 |

CNY, Chinese Yuan (US$1 = 7CNY); SD, standard deviation; IQR, interquartile range; BMI, body mass index; SBP, systolic blood pressure; DBP, diastolic blood pressure; FPG, fasting plasma-glucose; TC, total cholesterol; TG, triglycerides; HDL-C, high-density lipoprotein cholesterol; LDL-C, low-density lipoprotein cholesterol.
